# The Plegma dataset: Domestic appliance-level and aggregate electricity demand with metadata from Greece

**DOI:** 10.1038/s41597-024-03208-0

**Published:** 2024-04-12

**Authors:** Sotirios Athanasoulias, Fernanda Guasselli, Nikolaos Doulamis, Anastasios Doulamis, Nikolaos Ipiotis, Athina Katsari, Lina Stankovic, Vladimir Stankovic

**Affiliations:** 1 National Technical University of Athens, School of Rural, Surveying and Geoinformatics Engineering, Athens, 157 80 Greece; 2 Plegma Labs, Marousi, 151 24 Greece; 3 Aalborg University, Department of the Built Environment, Copenhagen, 2450 Denmark; 4 University of Strathclyde, Department of Electronic and Electrical Engineering, Glasgow, G1 1XQ UK

**Keywords:** Energy efficiency, Energy and behaviour, Technology, Energy management

## Abstract

The growing availability of smart meter data has facilitated the development of energy-saving services like demand response, personalized energy feedback, and non-intrusive-load-monitoring applications, all of which heavily rely on advanced machine learning algorithms trained on energy consumption datasets. To ensure the accuracy and reliability of these services, real-world smart meter data collection is crucial. The Plegma dataset described in this paper addresses this need bfy providing whole- house aggregate loads and appliance-level consumption measurements at 10-second intervals from 13 different households over a period of one year. It also includes environmental data such as humidity and temperature, building characteristics, demographic information, and user practice routines to enable quantitative as well as qualitative analysis. Plegma is the first high-frequency electricity measurements dataset in Greece, capturing the consumption behavior of people in the Mediterranean area who use devices not commonly included in other datasets, such as AC and electric-water boilers. The dataset comprises 218 million readings from 88 installed meters and sensors. The collected data are available in CSV format.

## Background & Summary

Over the past few years, there has been a growing adoption of smart meters on a worldwide scale. It is projected that the global market for smart meters will expand by 9% by the end of 2026, resulting in the replacement of conventional meters and contributing to the so-called smart grid transition^1^. For example, it is anticipated that 106 million smart electricity meters will be deployed in Europe between 2022 and 2027, primarily driven by large rollouts in the UK, Poland, Germany, and Greece, coupled with nationwide rollouts in various small and medium-sized European countries. As a result of these developments, it is expected that the installed base of smart meters in Europe will achieve 74% penetration by 2027^2^.

Smart meters offer a range of benefits that can improve the energy system for both consumers and energy providers. For energy providers, smart meters offer automated and accurate meter readings, enabling streamlined billing processes and better management of energy demand during peak periods. Meanwhile, for consumers, smart meters provide real-time information about energy usage, which allows them to make more informed decisions about their energy consumption, potentially leading to cost savings. There is a wide range of problems that can be addressed using smart-meter data, as highlighted by^3–5^. For the residential sector, smart meter data enable a variety of new applications and services, including appliance-level energy feedback^6,7^ enabled by non-intrusive load monitoring (NILM) approaches, where the appliance-level consumption patterns are extracted by exclusively analyzing the aggregate household energy consumption^8–13^; demand forecasting^14,15^; home energy management systems (HEMS) for home automation and energy conservation^16,17^; anomaly detection and retrofit recommendations, i.e., replacement of an energy expensive appliance^18–21^; demand side flexibility and load shifting^22–24^; ambient assisted living (AAL), i.e., providing useful insights into various health features by analyzing occupants’ consumption activity^25,26^. On this basis, electricity consumption datasets are crucial for the development and evaluation of signal processing and machine learning algorithms of such applications. To ensure the reliability and effectiveness of these algorithms, it is imperative that datasets be obtained from real-world settings, where households conduct their regular activities without any interference, as opposed to laboratory conditions or synthetic datasets. Such a methodology enables more accurate and effective testing of these methods, ensuring their reliability and usefulness in real-world applications^27^.

There are a number of open-source residential datasets^28,29^ that provide electric consumption data with varying char-acteristics; some of the most popular are presented in Table 1. These characteristics include measured electrical quantities (e.g., current, voltage, active power, apparent power), sensor placement (e.g., single point sensing, circuit level, and appliance level), campaign duration, campaign location (e.g., country, municipality), and metadata (e.g., building properties, user demo- graphics)^30^. Although all characteristics are important for determining the possible uses of each dataset, the presence of both aggregate and individual appliance measurements is crucial due to its vital impact on their potential usage, from NILM^31,32^ to appliance-level related applications such as understanding consumption patterns^33^ in life cycle analysis studies^34^ and mixed methods studies in relation to domestic practices^35^. Other datasets^36–38^ highlight aspects of occupant behavior, including details of occupant interactions with devices, equipment, and technical systems. These insights offer valuable information for enhancing the precision of energy simulations and occupant comfort in buildings.

**Table 1:** An overview of NILM datasets.

Dataset	Loc.	Duration, Year	No. Houses	Sensor placement	Data	Granularity	Metadata
IHEPCDS^65^	FR	47 months, 2006	1	Agg., 3-Sub.	P,Q	1 min	—
REDD^61^	USA	1 month, 2011	6	Agg., 9–24 App.	P,V	Agg. 15 KHz App. 1 Hz	—
BLUED^66^	USA	8 days, 2011	1	Agg.	V, I	Agg. 12 KHz	ON-OFF transitions
UK-DALE^62^	UK	655 days, 2015	5	Agg., 5–54 App.	V, I	Agg 16 KHz App. 6 sec	Building properties Occupants attributes
APMds2^67^	CA	2 years, 2012	1	21 App.	V,I,f Pf, P,Q S,E	1 min	Building properties Occupants attributes Ambient information
ECO^68^	CH	8 months, 2014	6	Agg., 6–10 App.	P,V,I, Q, Φ	1 Hz	Occupancy information
GREEND^69^	AT IT	1 year, 2014	9	9 App.	P	1 Hz	—
REFIT^27^	UK	2 years, 2015	20	Agg., 9 App.	P	8 sec	Building properties
DRED^70^	NL	6 months, 2015	1	Agg., 12 App.	P	1 Hz	Building properties Ambient information Occupancy information
IDEAL^71^	UK	23 months, 2020	255	Agg., App.	P	1 Hz	Ambient information Occupant information
IEDL^72^	IN	1 year, 2022	1	Agg., App.	P	1 min	Appliance information
SustDataED2^73^	PT	96 days, 2022	1	Agg., 18App	P,Q, V,I	Agg. 13 KHz App. 0.5 Hz	ON-OFF transitions
ECD-UY^74^	UY	21 days, 2022	110.953	Agg., 9 App.	P	Agg. 15 min App. 1 min	Occupants information Building properties

The table summarizes the country and year of the release for each dataset, the number of houses included, as well as the duration of the dataset, the measured variables, and the granularity and available metadata. Agg.  =  Aggregate, App.  =  Appliance, Sub.  =  Power circuit. Active Power (P), Reactive Power (Q), Apparent Power (S), Energy (E), Frequency (f), Power Factor (pf), Phase Angle (*φ*), Voltage (V) and Current (I).

**Fig. 1:**
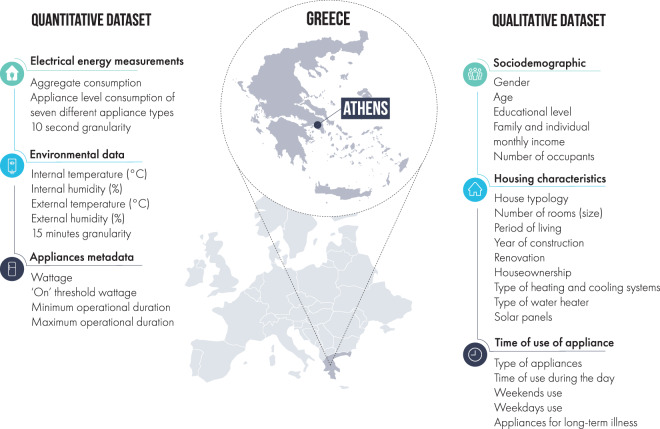
Plegma Dataset Overview.

The Plegma dataset^39^ (Fig. 1), presented in this paper, is the only public dataset providing residential electricity consumption measurements at a 10-second temporal resolution in Greece, and one of the first of its kind in the Mediterranean area. The dataset captures consumption patterns that are typical to the local climate and lifestyle, offering insights into understanding the characteristics of regional energy use. These insights are important for a deeper understanding of regional energy dynamics, enabling comparisons with other areas and aiding in the evaluation of the transferability of energy-related applications across various settings. Comprised of both quantitative and qualitative components, the Plegma dataset offers a dual perspective on energy use. The quantitative section encompasses aggregate household consumption and itemised appliance-level data from 13 distinct households. This includes data on specific appliances like air conditioners and electric water boilers, which are less commonly recorded in other datasets. Additionally, the Plegma dataset incorporates measurements of both internal and external environmental parameters, specifically temperature and humidity. The qualitative data cover details on sociodemographics, building characteristics, and patterns of appliance usage; crucial information for energy flexibility strategies. Data collection of the Plegma dataset^39^ started in July 2022. The collected data are recorded at a sampling rate of 10 seconds in order to be similar to the specifications of SMETS2 HAN^40^ available recommended resolution and to ensure the real-world applicability of the developed solutions, which are based on our dataset. This sampling rate mirrors the granularity chosen by other prominent datasets, such as REFIT and UK-DALE, which are sampled at 8 seconds and 6 seconds, respectively, underlining a standardized approach to granularity in datasets of this nature. Other datasets, such as REDD, BLUED, and SustDataED2, have a high-frequency sampling rate (over 10 kHz), but they are recorded only for a few weeks and include a very limited amount of houses. Others, such as AMPds, IHEPCDS, IEDL, and ECD-UY, have been recorded at a lower temporal resolution of 1 min or less, limiting their usability for high-frequency applications^9^.

Following the suggestions of^30^, which provides recommendations for electricity consumption datasets collection, storage, and provision, we ensure the Plegma dataset’s interoperability and comparability. Some of the most important recommendations that the Plegma dataset addresses are the *campaign duration*, which lasts for more than a full year, enabling capturing gradual changes in appliance usage patterns (e.g., due to seasonal changes or human behavior), *sensor placement* which follows a deployment of sub-metering devices providing consistent data for each of the monitored houses, *metadata* which provide a variety of demographic data and building characteristics, *file format* which provides a comma-separated values (CSV) format that is widely used to store similar data and is also compatible with NILM tools and energy analytics algorithms, and *accessibility of dataset* which is open access and easily accessible through Strathprints, the University of Strathclyde’s institutional repository. In addition to the aforementioned recommendations, the Plegma dataset^39^ also rigorously aligns with the FAIR data principles^41,42^, embodying the core research integrity values of honesty, cooperation, reliability, and accountability. Findability of the dataset is ensured through standard DOI identification and is easily discoverable through a standard search engine. Regarding Accessibility, the dataset is hosted in the University of Strathclyde’s repository, guaranteeing open access along with essential documentation and open access code hosted on the project’s GitHub page. The use of the CSV file format and the dataset’s file organization (Fig. 5) and available metadata description strengthens Interoperability, allowing data exchange and reuse across various NILM tools and energy analytics algorithms. The inclusion of explicit licensing terms, such as Creative Commons, alongside comprehensive documentation on the required software for accessing and utilizing the datasets, enhances the dataset’s Reusability aspect.

To address the ethical considerations and consent procedures associated with our data collection, we followed protocols that ensure the respect and privacy of all participating households. The study also received ethical approval from the National Technical University of Athens’ ethical committee (https://www.elke.ntua.gr/en/ethics-committee/), under the file number 15623. Participation in this study was entirely voluntary, with comprehensive informed consent obtained from each household prior to data collection. This process involved detailing the study’s purpose, the nature of the data to be collected, and the use of such data for research and innovation within the scope of the European Commission-funded Marie Skłodowska-Curie Action GECKO project. Participants were informed that they could withdraw at any time without any consequences. All collected data were anonymized to protect participant privacy, with personal identifiers removed and stored securely within Plegma Labs’ databases. This approach underscores our commitment to ethical research practices and the safeguarding of participant rights and privacy.

## Methods

### Selection methodology

The houses participating in data collection include households that are part of the Athenian community, which constitutes a newly established non-profit energy community in the municipality of Attika in Greece. The community was established as an initiative by a group of technology and engineering professionals with many years of experience in research and development. The selection of households for the Plegma dataset was not confined to households deeply versed in these fields but extended to those with a fundamental acquaintance with information and communication technology (ICT). This foundational understanding is characterized by the presence of an internet connection and a readiness to adopt the suggested energy monitoring setup. Additionally, the dataset encompasses a diverse range of demographics, from retired and working couples to families and single-person households, highlighting the relevance and applicative value of our dataset across a broad spectrum of households.

To enhance user engagement in data collection, the developed energy monitoring system was extended to incorporate an intuitive graphical user interface. This application helps participants to monitor and visualize their energy consumption in real-time, providing a powerful incentive for their participation. Some of the homes were not included in the data collection process, primarily due to connectivity issues. These issues include underground utility meters, which make signal acquisition challenging, or architecture-related obstacles, such as thick walls or metal objects that weaken or block the Z-wave signals^43^. The selected houses’ occupancy information, physical characteristics, and heating system are presented in Table 2.

**Table 2:** An overview of the houses included in the study.

House	Occupancy	Dwelling age	Dwelling type	Size (#rooms)	# Monitored Appliances	Electric Phase	Heating type
1	1	1970	apartment	3	5	single-phase	Radiator oil
2	1	1965	apartment	3	5	single-phase	Electric heater
3	1	1970	apartment	4	5	single-phase	A/C
4	3	2000	apartment	4	5	single-phase	Electric heater
5	3	1980	detached house	4	5	three-phase	Radiator oil
6	2	1960	apartment	4	3	single-phase	Radiator gas
7	1	1980	apartment	4	5	three-phase	Air-to-air heat pump
8	1	2010	apartment	3	4	single-phase	Underfloor heating
9	1	1960	apartment	3	3	single-phase	Electric heater
10	4	2000	apartment	3	6	single-phase	Radiator oil
11	3	1965	apartment	4	7	single-phase	Radiator oil
12	2	1985	apartment	5	6	three-phase	Radiator oil
13	1	1980	apartment	3	4	single-phase	Radiator gas

The “Occupancy” column indicates the total number of individuals residing in the home during the observation period. The column labeled “Number of Monitored Appliances” displays the total count of electrical devices in the home from which data is being gathered. Additionally, the number of rooms provides information on the size of each residence. The primary heating system used in each house is detailed in the Heating type column.

In each house, both the aggregate and appliance-level consumption were monitored. Appliance selection was motivated by incorporating appliances with relatively high electricity consumption that are also commonly used in Greece and other countries with similar environmental conditions. The Plegma dataset encompasses data on AC units, which are widely utilized in Greek households for both heating and cooling purposes. These units are split type, installed individually in each room. Another important appliance type featured in our dataset, and also widely used in Greece and other Mediterranean countries, is the electric water boiler, used for domestic hot water. The Plegma dataset also includes frequently used appliances such as refrigerators, washing machines, and dishwashers, which are also presented in the majority of the electric consumption datasets. Table 3 presents a detailed list of the appliances monitored in each household, while the dataset also provides information regarding their wattage (i.e. power drawn by the appliance), the minimum threshold for an appliance to considered as ‘on’, the requisite minimum duration for an appliance to be categorized as active and the requisite minimum duration for an appliance to be categorized as inactive. All this information can be used as a guideline to help the dataset users assess the potential for transferability to their use cases and further refine their analysis by being able to determine the operational status of each specific appliance.

**Table 3:** Monitored appliances in each house.

Appliance	House ID													
	1	2	3	4	5	6	7	8	9	10	11	12	13	total
Washing Machine	✓	✓	✓	✓	✓	✓	✓	✓	—	✓	✓	✓	✓	12
Dishwasher	—	—	—	—	✓	—	—	✓	—	✓	—	✓	—	4
Air Conditioner	2 ✓	✓	2 ✓	2 ✓	✓	—	2 ✓	✓	—	2 ✓	3 ✓	✓	✓	18
Fridge	✓	✓	✓	✓	✓	✓	✓	✓	✓	✓	✓	2 ✓	✓	14
Boiler	✓	✓	✓	✓	✓	✓	✓	—	✓	✓	✓	✓	✓	12
Washer Dryer	—	—	—	—	—	—	—	—	✓	—	—	—	—	1
Kettle	—	✓	—	—	—	—	—	—	—	—	—	—	—	1

The final column shows the total number of the same type of appliances.

### Data collection setup

**Fig. 2:**
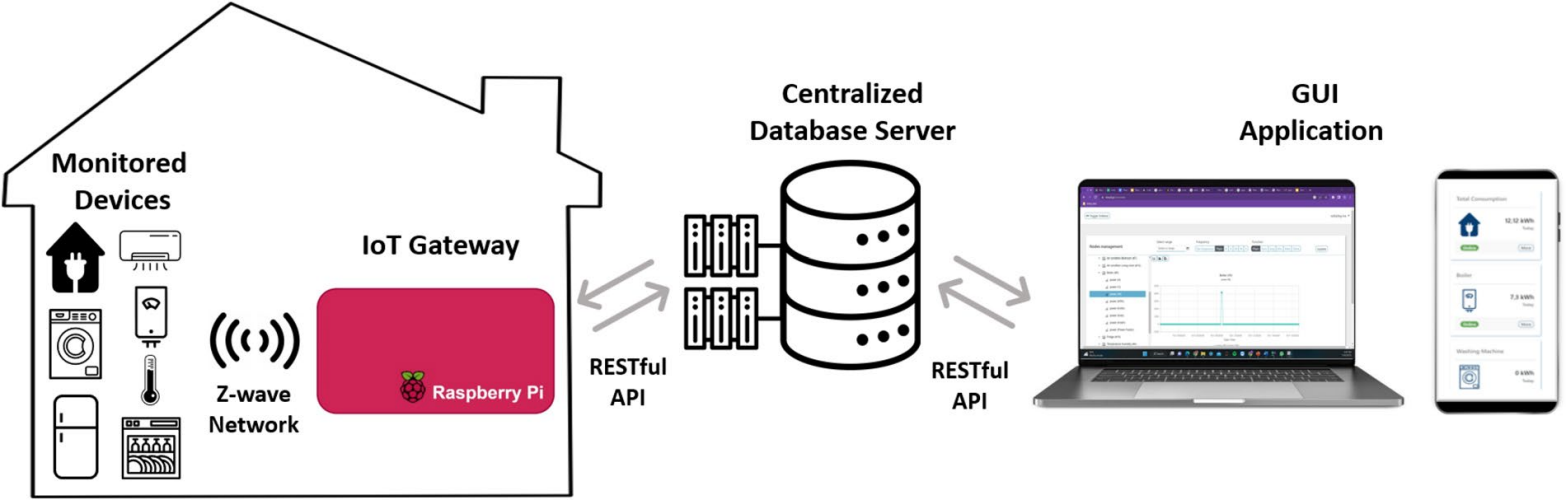
Overview of the developed energy monitoring and collection framework.

To facilitate the data-gathering process, an end-to-end energy monitoring and data collection framework was developed. An overview of the developed data acquisition system is shown in Fig. 2, whereby monitored devices communicate with the IoT gateway and transmit data every 10 seconds. To ensure dependable, scalable, and high-performing equipment for the data collection and monitoring system, commercially available hardware from reputable home automation companies such as Aeotec and Qubino was used. Raspberry Pi was selected as the IoT gateway for the data collection and monitoring system, as it is widely recognized for its suitability and versatility in home automation solutions^44–46^.

The overview of the developed energy monitoring framework can be depicted in Fig. 2, which comprehensively illustrates all the different stages of the data acquisition process. Firstly, The monitored devices communicate with the IoT gateway through the Z-Wave communication protocol. Subsequently, the gateway forwards the data to a central database server, AMD 2nd generation EPYC CPUs (8 VCPU, 16 GB RAM), and stores them in a PostgreSQL for secure preservation, leveraging a RESTful API. Finally, the collected data can be accessed through the developed GUI application, which directly communicates to the database server via RESTful API calls. Our solution utilizes the Z-Wave communication protocol, providing a stable interaction between the monitored devices and the gateway. The basic reason for selecting the Z-Wave protocol is that it was developed primarily for use in connected home technology, and it is also recommended for the SMETS2 ecosystem^40,47^. Furthermore, compared to other wireless technologies like Bluetooth and Zigbee, it provides higher reliability and coverage^48–50^. Finally, Z-wave stands out for its power efficiency relative to WiFi devices, where they consume much more power and can pose serious drawbacks for battery-powered devices such as environmental sensors^51^. The majority of the advantages of this technology stem from Z-Wave’s utilization of a mesh network topology^43^, which allows devices to communicate through a series of nodes. Specifically, every Z-Wave device within the network can act as a repeater, extending the network’s range and reliability as they enable them to find alternative paths to communicate if one path is blocked or unavailable^47^. To gather the data from the 13 houses, we employed 13 IoT gateways and 88 sensors and smart meters, comprising 62 appliance smart plugs as shown in Table 3, 13 environmental sensors (1 per house) and 13 smart meters to measure the total aggregate consumption (1 per house).

### IoT Gateway

**Fig. 3:**
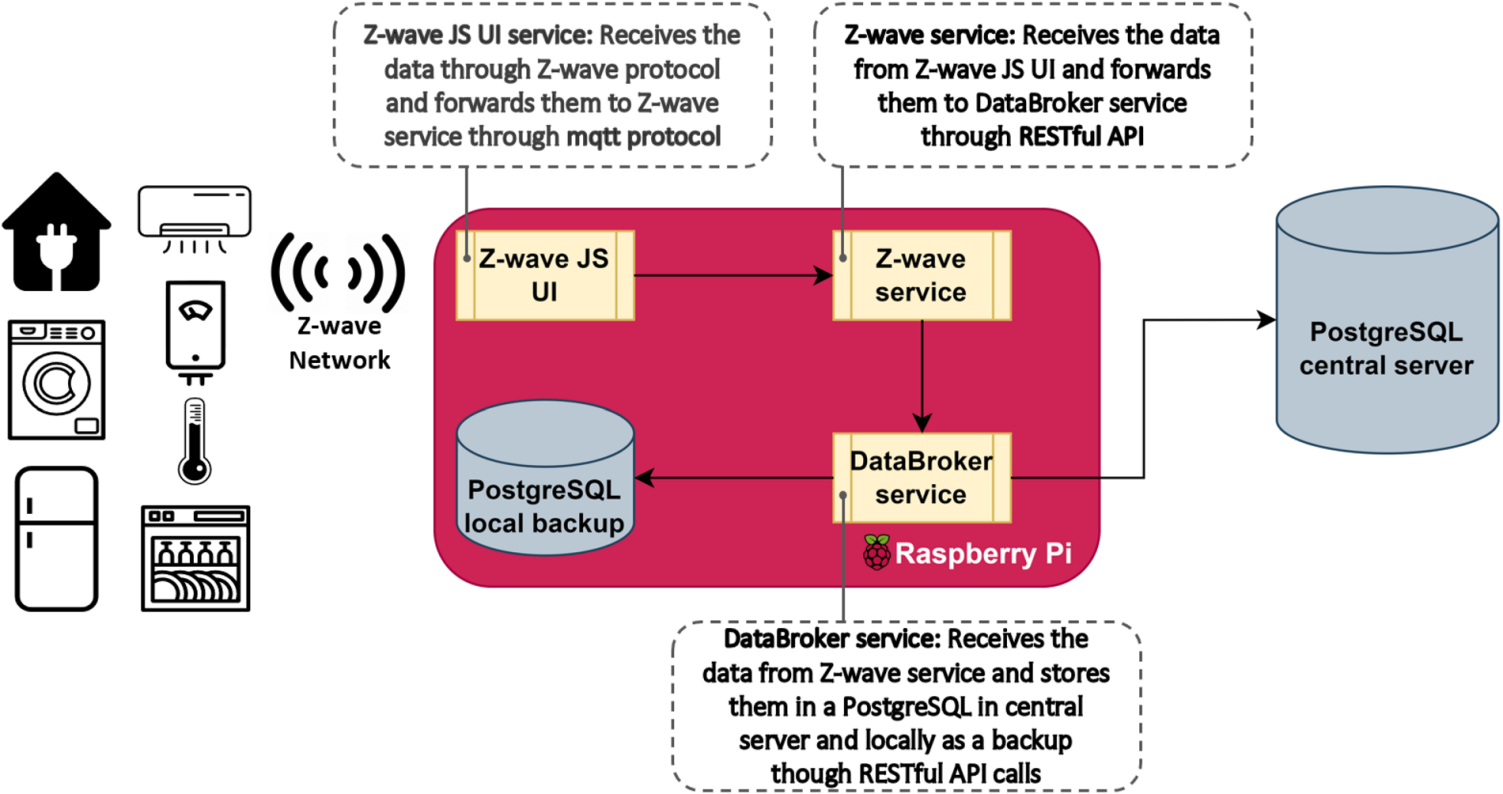
Overview of the developed IoT Gateway software services.

One of the most important components of the developed data collection and monitoring framework is the IoT gateway, which acts as a bridge between the monitored devices and the central server. Specifically, it consists of a Raspberry Pi Model 4 that is equipped with an Aeotec Z-Pi7 Z-wave daughter card. The inclusion of the Z-wave daughter card enables the gateway’s communication with the Z-wave devices, allowing it to receive and collect consumption and environmental data. These data are first stored in a local PostgreSQL database, and they are subsequently forwarded to the central server. This configuration eliminates the data gaps in the collection process since it allows our system to persist the data collection even in the case of a network disruption or internet connectivity failure, which would otherwise prevent the gateway from transmitting the data. To enable the data collection process described above, the appropriate software stack was developed, comprising three distinct modules as illustrated in Fig. 3. A detailed description of the role and functionality of each service is provided below.

- **Z-wave JS UI service** (code available at https://github.com/zwave-js/zwave-js-ui) is an open-source soft- ware that enables developers to build IoT applications utilizing the Z-wave communication protocol. This service is also equipped with a user interface (UI), which is a web-based application that enables users to configure and administer their Z-wave network in an intuitive and easy way. The developed framework employs a dockerized version of the service, which provides a secure and easily deployable solution. The basic functionality of the Z-wave JS UI service is to communicate with the monitored devices utilizing the *Z-wave communication protocol* and forward the collected data to the Z-wave service.
- **Z-wave service**(code available at https://github.com/sathanasoulias/Plegma-Dataset/tree/main/data_collection) is a service developed in Node.js and includes the main functionalities of the IoT gateway. Its main role is to obtain the collected data from the Z-wave JS UI service via *MQTT communication protocol* and forward them to the DataBroker service. MQTT is a lightweight communication protocol that has been widely utilized in many IoT solutions since it is intended to work under low bandwidth on low-power machines^52,53^. Furthermore, this service is also responsible for (1)- determining which data are going to be forwarded to the DataBroker service, (2)- managing the configuration file of the monitored devices, which can be used to restore the system in case of hardware failure, and (3)- handling actuation commands to facilitate the communication between the server and the devices. Z-wave service promotes the framework’s interoperability by providing system parameterization functionalities and enhances its flexibility and compatibility with other solutions.
- **DataBroker service** (code available at https://github.com/sathanasoulias/Plegma-Dataset/tree/main/data_collection) is a service developed in Node.js, and it is responsible for receiving the collected data from the Z-wave service and verifying their format structure before transmitting them to the central database server. DataBroker service is also responsible for keeping local backups of the collected data to a secondary PostgreSQL database located in the IoT gateway, avoiding data gaps in case of network failure. The communication between both the local PostgreSQL database and the central PostgreSQL database server is executed using *RESTful API calls*.

### Household aggregate consumption

Whole-house consumption data has significant value since it emulates the data that will be recorded by smart meters in the near future and can be used to build applications in various domains such as demand forecasting, demand response, detection of activities of daily living, or energy disaggregation. The household aggregate consumption was measured using the Aeotec Home Energy Meter (https://aeotec.com/products/aeotec-home-energy-meter/), which contains a single phase current clamp and a Z-Wave transmission module that transfers readings every 10 seconds using a Z-Wave frequency 868 MHz to the Z-wave JS UI service of the IoT gateway.

Aeotec Home Energy Meter has been successfully used in many IoT-based solutions^54–56^ as it provides robust wireless connectivity with a range of 150 meters as well as secure broadcasting with AES-128 encryption protocol. Furthermore, it can record up to 200 amps with 99% accuracy in real-time, making it an ideal solution for home energy monitoring. Finally, another reason for our choice is that Aeotec Home Energy meters operate under the Z-wave communication protocol, which exhibits superior reliability and broader coverage compared to other wireless protocols^51^. The monitored measurement readings included Watt (W), Volt (V), Ampere (A), and kilowatt hour (kWh).

Three houses in the study had three-phase power. In these three cases, a three-phase version of the Aeotec Home Energy Meter was installed, which contains 3 clamps, one for each phase. The rest of the process follows the same configurations, collecting readings from the same measurements with a 10-second granularity.

### Individual appliance consumption

For the purpose of the study, every household was equipped with a suitable number of smart plugs designed to gather consumption data at the appliance level. Each smart plug provided readings of the active power (W) of each selected appliance reported every 10 seconds.

The data collection process of domestic devices such as washing machines, dishwashers, A/Cs, and refrigerators was facilitated by Aeotec Smart Switch 6 plugs (https://aeotec.com/products/aeotec-smart-switch-6/). Smart Switch 6 plugs operate under the Z-wave communication protocol, are easy to install, and can experience an error of at most 1%. For the water boiler, which is a high load-consuming device and has never been included in any similar dataset, a Qubino smart meter (https://qubino.com/products/smart-meter/smart-meter-techold/) was used since it can operate up to 65 A in a Z-Wave frequency of 868.4 MHz.

The readings from all the monitored devices were transmitted to the IoT gateway utilizing the Z-wave communication protocol and the Z-Wave JS UI service. Subsequently, these data were forwarded to the central database server for storage and further pre-processing. The recording of the appliances started during the initial installation of the equipment, and recruited households were given specific instructions to avoid unplugging or repositioning the smart plugs during the data collection period.

### Environmental data

The environmental data included in the Plegma dataset encompass both internal and external temperature (°C) and humidity (%). The data collection of the internal environmental data was conducted using the Multisensor 6 (https://aeotec.com/products/aeotec-multi-sensor-6/), which operates under the Z-wave communication protocol and presents an accuracy of ±1 °C for the temperature and ±3%RH for the humidity data. The indoor environmental data were recorded every 15 minutes and were forwarded to the central database server through the IoT gateway.

The outdoor environmental data were collected using the open API that provides historical weather data produced by MET Norway (https://api.met.no/weatherapi/), and they are available under a Creative Commons license. The dataset includes both the external temperature and humidity with a 1-hour granularity.

### Server & database

The consumption data from all the monitored appliances, together with the whole-house consumption and the internal environmental data from all the houses participating in the survey, are stored in a PostgreSQL server hosted by Hetzner, in Nuremberg, Germany, with the following specifications: AMD 2nd generation EPYC CPUs (8 VCPU, 16 GB RAM), running on Ubuntu 20.04.5 LTS (GNULinux 5.4.0-131-generic x86_64) with PostgreSQL 14.5.

The operation of monitoring the health status and connectivity of the monitored devices was carried out using a developed Javascript script. This script evaluated the temporal difference between the last recorded measurements in the database and, in the case of a significant time gap, in excess of 30 minutes, an automatic email was sent to flag the issue for further investigation. The health status of the monitored devices was also available through the developed UI application.

### Sociodemographic, building characteristics and appliance usage metadata

A baseline questionnaire on participants’ sociodemographic aspects and building characteristics was developed to provide an overview of social and building factors that may impact energy consumption, such as number of occupants, age, educational background, gender, income, house typology, size of the house, heating and cooling systems, among others. Furthermore, following Trotta *et al*.^57^, questionnaire on the time of use of electric appliances, detailed data on participants’ habits and routines in relation to the use of appliances were collected for further scrutiny of variation and patterns of such activities, which are related to energy demand and flexibility. Both questionnaires were applied to all 13 participants in the summer of 2023 via phone (n = 4) and face-to-face (n = 9), primarily in English but also in Greek when necessary. The first author conducted the data collection by asking the questions and filling out the questionnaire, a procedure that lasted, on average, 20 minutes.

### Data pre-processing pipeline

The collected electric and environmental data were pre-processed and cleansed in order to be used as a common baseline for researchers using the Plegma dataset^39^. The pre-processing was carried out using Python language version 3 and several libraries, including Pandas, Numpy, and Plotly for visualizations. The basic components of the proposed data processing pipeline are described in the following sections. The developed code for visualizing and preprocessing the dataset can be found in the project’s GitHub page (https://github.com/sathanasoulias/Plegma-Dataset).

#### Data synchronization

**Fig. 4:**
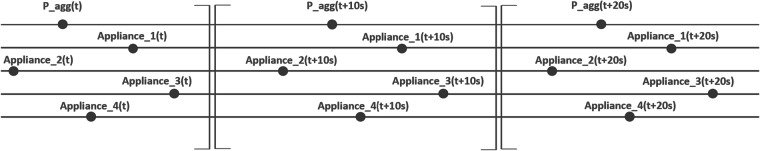
Overview of synchronization issue. Each line represents a sensor, P_agg being the whole-house consumption. The dots represent each recorded measurement, and t is the timestamp when the corresponding data point was received at the IoT gateway.

The installed monitoring devices (energy meters, smart switches, and environmental sensors) were only capable of broadcasting their readings, which resulted in the readings not being synchronized with each other as shown in Fig. 4. The timestamp assigned to each measurement was the UNIX timestamp when the corresponding data were received at the IoT gateway.

The synchronization of the different measurements was achieved through the technique of resampling, a common method used in temporal data analysis. Resampling involves altering the frequency of the data samples to align them in a common time vector^58^. In this context, resampling involved aligning the various electrical and environmental time series to a common time vector, thereby synchronizing their measurements at consistent 10-second and 15-minute intervals correspondingly. Let *X* (*τ*) denote the original time series where *τ* signifies the time index. Resampling aims to transform *X* (*τ*) to *X*′(*τ*′) where *τ*′ represents a regular grid of 10-second intervals. For every timestamp *τ*′ in the new time vector, the corresponding value of *X*′(*τ*′) was estimated based on the values *X* (*τ*) closest to *τ*′. Thus, the relationship between the resampled and original time series values can be formally expressed as *X*′(*τ*′) = *X* (*τ*), where *τ* is the timestamp in the original time series that is closest to *τ*′. This procedure resulted in a set of electrical (aggregate and appliance level) and environmental (temperature and humidity) time series that were harmonized to the same time vector, facilitating synchronized analysis on a uniform time grid.

#### Abnormal measurements

In the analysis of the collected data, sporadic discrepancies were identified. Specifically, these inconsistencies manifested as unexpected spikes and measurements that exceeded the standard power thresholds of the monitored devices and the ambient environmental conditions. Potential explanations for these inaccurate readings could be electrical interference since IoT devices can be affected by other electronic devices in close proximity, leading the sensor to log unexpected spikes or abnormal values. Additionally, software glitches in the IoT sensor or imprecise calibration and built-in offset inaccuracies can be some other contributing factors to observed erroneous measurements. Nonetheless, these wrong readings comprised a mere 1.65% of the total measurements. To address the issue of atypical measurements, the detected abnormal data points were replaced with the preceding normal reading.

##### Algorithm 1

Allocation of intersecting areas - Pseudocode.

A reading is deemed ‘normal’ if it is found within the expected range. For electrical data, the thresholds for a value to be considered as abnormal includes a tolerance margin to the appliance standard wattage since some devices may have a large instantaneous surge when they are turned on. Additionally, we verify the accuracy of a measurement by comparing the total wattage of individual appliances against the reading from the central smart meter. If the combined appliance wattage exceeds the smart meter’s reading, especially when an appliance’s wattage measurement surpasses its threshold, this serves as an indication of an abnormal value. For environmental metrics, acceptable temperature ranges are set from 10 °C to 50 °C, and humidity values are confined between 0% and 100%. However, acknowledging the variety of techniques for detecting abnormal readings, we have provided access to both the raw dataset and the appliance metadata information to enable users to employ their chosen methods for processing and analyzing the data.

##### Algorithm 2

Interpolate Consecutive Short Data Gaps.

#### Data gaps

The dataset contains intermittent gaps in consumption and environmental readings, typical in large-scale data collection, presenting analytical challenges. Several potential reasons can underpin these discrepancies. Firstly, Z-Wave operates on specific frequency bands, which can occasionally face interference from other devices or electronic noise. Secondly, physical obstructions or the presence of certain materials in the communication path can attenuate the Z-Wave signals, leading to dropped packets. Additionally, network congestion due to a high number of devices communicating simultaneously or firmware issues in the Z-Wave devices themselves can result in data losses. To ensure the integrity of the subsequent analyses and make the dataset compatible with applications like NILM, where granularity and continuity of data are essential, a rigorous data imputation methodology was implemented. For the electrical energy measurments, gaps less than 30 seconds were interpolated, whereas in the environmental data, gaps were filled only if they were shorter than 1 hour. Interpolating short data gaps ensures accuracy due to the closeness and continuity of adjacent data points. These interpolated values thus likely reflect genuine readings, safeguarding data integrity. Conversely, larger gaps introduce greater uncertainties. Spanning a more extended period, their interpolation risks generating values that deviate from actual scenarios. Especially in applications like NILM, this can amplify errors during load disaggregation or pattern analysis. Hence, to preserve data reliability, these larger gaps were left unaltered.

## Data Records

The Plegma dataset^39^ is accessible in both raw and clean formats, and the collected data are provided as comma-separated values (CSV) files at Strathprints, the University of Strathclyde data repository. The unprocessed dataset encapsulates the originally collected data, unaltered by any preliminary processing, thereby encompassing abnormal values and data gaps attributed to equipment malfunctions. The primary rationale for offering this raw version of the dataset is to give potential users the opportunity to carry out their individual pre-processing techniques.

**Fig. 5:**
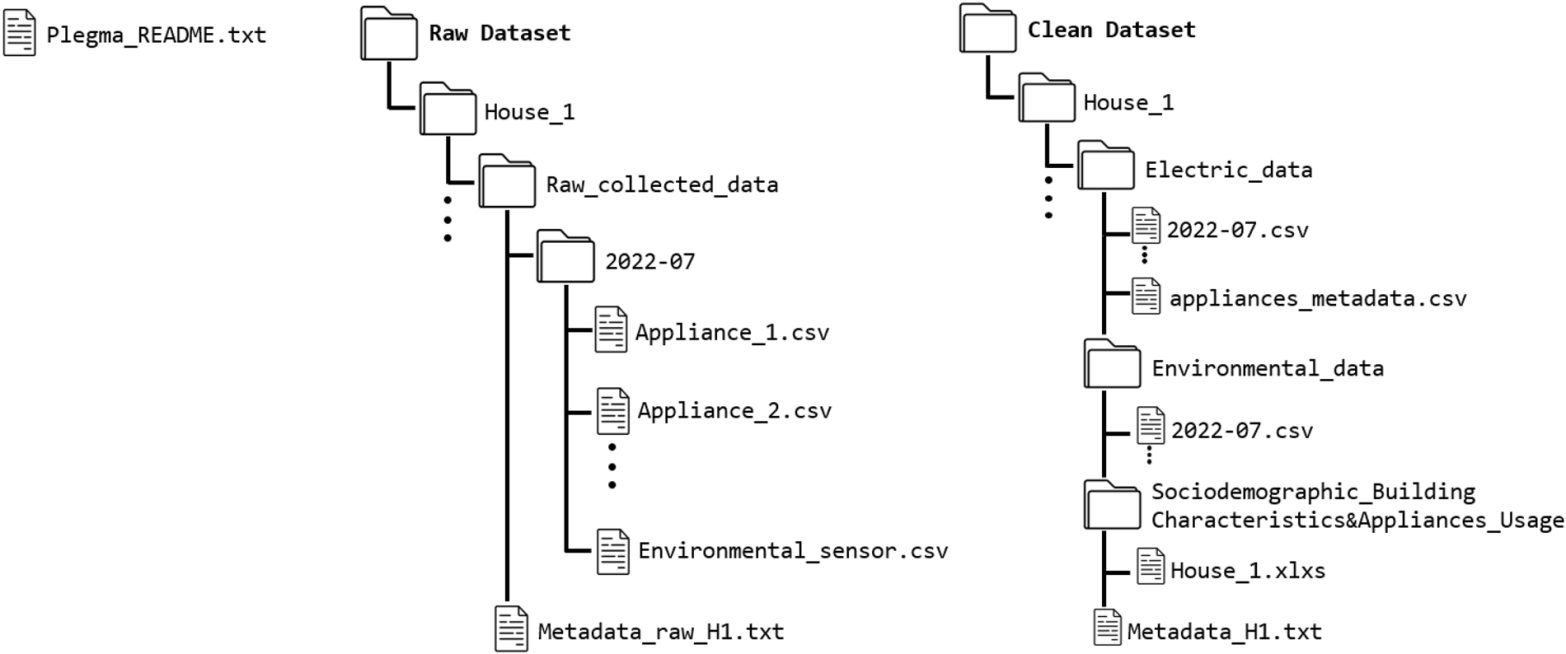
Overview of the dataset’s folder structure.

The clean version of the dataset, in contrast, represents the dataset after the implementation of a proposed pre-processing pipeline. This pipeline addresses several key components, including i) the standardization of units, date formats, and column name conventions, ii) the handling of abnormal measurements and data gaps due to failure of the equipment or internet connectivity disruptions, iii) the data synchronization, and iv) the identification and flagging of known issues, timestamps where the appliance level reading exceed the monitored total consumption due to synchronization mismatch, instrumental accuracy or inductive and capacitive appliance-level loads. The folder structure of the Plegma dataset is presented in Fig. 5. Both Raw Dataset and Clean Dataset directories contain 13 sub-directories named House_* < i > *, where i is an integer between 1 and 13. In the Raw Dataset, each house sub-directory contains a Metadata_raw_H < i > .txt file with the house metadata (monitored appliances & recorded values) and the unprocessed collected data organized in each folder per month, which contains the collected data from the installed smart meter, smart plugs, and environmental sensor in CSV formats. In the Clean_dataset folder, each house directory contains three sub- folders for the Electric_data, Environmental_data, as well as Sociodemographic_Building_Characteristics& Appliance_Usage along with the house metadata metadata_H < i > .txt, which contains the same information as the ones presented in the raw version of the dataset.

### Electrical energy measurements

Within the cleaned version of the Plegma dataset, the processed consumption metrics for each house are located within the Electric_data directory. Corresponding to each month, a CSV file is structured according to the nomenclature year-month.csv and encompasses the fields delineated in Table 4. In addition to these electrical data points, The directory appliances_metadata.csv provides specifics for each appliance, including wattage, minimum active duration (“min_on”), and minimum inactive duration (“min_off”).

**Table 4:** Description of the records in files of the electric consumption data.

Electric_data/year- month.csv		
field	*type*	*description*
datetime	string	Datetime of the record in MM/DD/YYYY HH:MM:SS AM/PM format (UTC + 3)
V	float	Instant voltage in Volts (V)
A	float	Instant current in Amperes (A)
P_agg	float	Instant aggregate power in watts (W)
appliance_1 . . appliance_n	float	Instant appliance power in watts (W)
issues	integer	Timestamps where the reading at the appliance level surpasses the total monitored consumption are marked as 1, while all other instances are flagged as 0.

#### Environmental data

The processed environmental data of each house are available in the corresponding Environmental_data folder. For each month, there is a CSV file named using the year-month.csv convention, similar to the organization of the consumption data, and includes the fields described in Table 5.

**Table 5:** Description of the records in files of the monitored environmental data.

Environmental_data/year- month.csv		
field	*type*	*description*
datetime	string	Datetime of the record in MM/DD/YYYY HH:MM:SS AM/PM format (UTC + 3)
internal_temperature	float	Instant internal temperature in Celsius
internal_humidity	float	Instant internal humidity (%)
external_temperature	float	Instant external temperature in Celsius
external_humidity	float	Instant external humidity (%)

### Sociodemographic building characteristics & appliances usage

The sociodemographic, building characteristics and appliance usage data can be found in the corresponding folder of each house in a Microsoft Excel Worksheet (.xlsx) format, which is structured in three different subsections, one for each category.

**Fig. 6:**
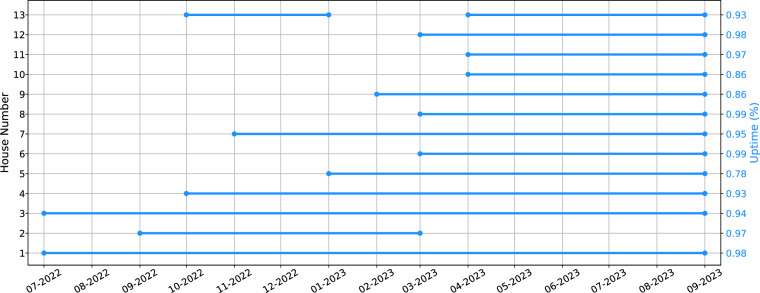
Plegma Data Availability. The left y-axis represents the corresponding house number, while the right y-axis represents the uptime of the corresponding data collection campaign.

**Fig. 7:**
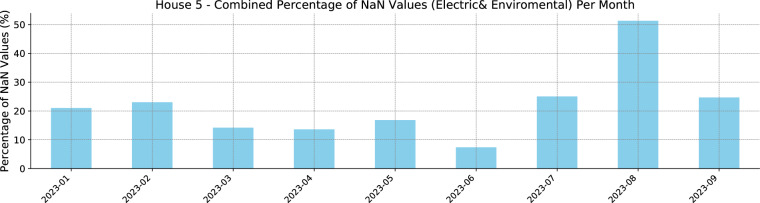
Combined percentage of NaN values (Electric& Environmental) per month for House 5.

The sociodemographic section includes the gender, occupation, educational level, age, number of occupants, and income information; the building characteristics include information such as house topology, number of rooms, and construction year, while the time of use of appliances present information on how often and what time of day each appliance is being used according to the perception of the occupants that can be used as soft labels. The data availability for all the included Plegma houses can be seen in Fig. 6. The vertical right edge shows the uptime per house, showing the percentage of the non-missing values for both electric and environmental data per house during their corresponding data collection campaign period. The average uptime across all houses was 93.44%, with House 5 having the lowest at 78.38% and House 6 the highest at 99.37%. For the calculation of the uptime percentages, both environmental and electrical data were considered, noting that data for both these categories do not necessarily miss synchronously. Regarding House 5, which displays the lowest percentage of non-missing values at 78%, the significant amount of missing data can be largely explained by certain technical difficulties unique to this residence. Firstly, the apartment’s structure featured thicker walls and numerous built-in devices, which complicated the establishment of an effective network topology for the Z-Wave system^50,59^. Additionally, the house’s occupants were away for an extended period in August 2023. During their absence, the house’s central electricity supply was switched off, resulting in the disruption of data collection and a loss of nearly half the data for that month, as seen in Fig. 7.

Finally, a Plegma_README.txt file is also available in the root level of the dataset, providing information regarding the folder structure, granularity, naming convention, and metadata.

## Technical Validation

### Electric & environmental data

Over the course of the data collection campaign, a total of 218,410,245 readings marked with timestamps were recorded, encompassing both electrical and environmental measurements from each of the 13 houses involved in the project. Out of these, 6.86% were identified as Not a Number (NaN) values, indicative of instances where our system failed to retrieve the requested data successfully. While this percentage might initially appear substantial, it is in fact similar to NaN values in datasets of this nature^60^.

For example, the REFIT dataset^27^, which is among the most referenced in this area, shows a close percentage of NaN values at 6.4%. This similarity suggests that the presence of NaN values is a standard aspect of data collection in such environments, arising from the complexities involved in long-term data acquisition^50,59^. In the electrical dataset, an ‘issues’ column has been incorporated to signify instances where the cumulative readings from individual appliances surpass the recorded aggregate consumption for a given sample. This condition is denoted by a value of ‘1’; conversely, a ‘0’ indicates normal readings. However, the proportion of timestamps flagged with 1 in this manner within the dataset amounts to only 0.82%. As previously noted, the main factors causing this issue are related to synchronization mismatches and deviations pertaining to instrumental accuracy. All the collected data, including electrical and environmental, has been visually inspected to verify the quality of their signatures. In all of the houses folders, the corresponding metadata_H < i > .txt file indicates the data availability in terms of installed sensors in each specific house. The quality of readings for certain appliances is impacted by their placement or disruption from nearby devices. This effect is particularly pronounced for appliances that are situated farther away from the IoT gateway, as well as for sensors that are installed behind large appliances like washing machines and fridges.

**Fig. 8:**
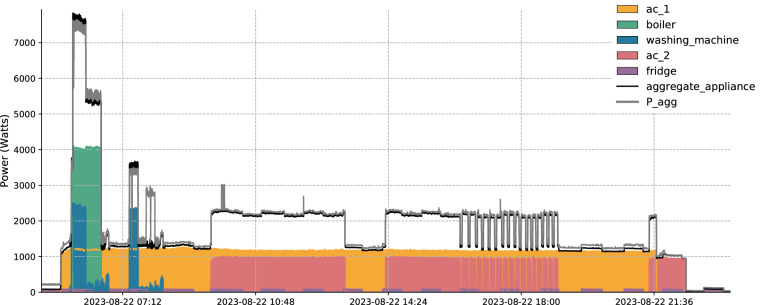
The power usage for House 1 on the 22nd of August 2023. The space between the aggregate_appliance consumption curve and the total power consumption (P_agg) curve illustrates the energy consumed by appliances that are not under monitoring.

Previously, we mentioned that the installed sensors did not report values synchronously with the smart meter of the aggregate consumption. Specifically, the appliance level measurements could differ by two or even three readings. However, after the synchronization pre-processing step described in the previous section, all the readings were harmonized under the same time vector. Figure 8 shows that even though this discrepancy occurs during the data collection process, the data signatures are well synchronized since the moments when appliances are turned on or off can be distinctly seen in the aggregate readings. Table 6 shows the percentage of total household consumption captured by sub-metering across the 13 houses involved in the project. According to this table, the average percentage of sub-metered consumption is 54%, while in some cases it can go up to 85% as happens in house 1. The low percentage of sub-metered energy consumption observed in some houses reflects the absence of monitoring for certain high-consumption appliances like ovens and stoves. The main reason that we decided to exclude these types of appliances from our dataset was the increased complexity involved in the installation process of the monitoring equipment to built-in devices. Although the magnitude of this gap may appear substantial, it is important to note that similar percentages have been documented in other highly cited datasets^27,61,62^, which have been extensively used across various research projects. The appliances chosen for sub-metering in our study are specifically those characterized by high energy consumption and flexible loads, thus making a significant contribution to the household’s total power peak, aligning with the objectives of our research to identify key areas for energy efficiency improvements and demand response initiatives.

**Table 6:** Household energy consumption, sub-metered consumption, and percentage of sub-metered consumption per house.

Household Energy Consumption and Submetering Statistics													
*House ID*	1	2	3	4	5	6	7	8	9	10	11	12	13
Aggregate Consumption (kWh)	2958	1415	2954	2818	3778	1512	2045	1222	1218	1544	2861	3664	3073
Sub-metered consumption (kWh)	2534	701	1557	1728	2356	744	633	574	470	919	2154	977	2128
Percentage of sub-metered consumption (%)	85	49	52	61	62	49	30	47	38	59	75	26	69

Finally, Table 7 shows the amount of the total recorded consumption and activations among the different appliance categories. This information could be important for applications such as NILM, where it is important to capture a large amount of appliance uses. NILM requires comprehensive training datasets encompassing a wide range of appliance types to develop accurate models^9^. By detailing the total recorded consumption across various appliance categories, our dataset provides researchers with critical insights into its potential applicability for training NILM models. This information enables researchers to assess how our dataset could support the testing and validation of their models under diverse scenarios, thereby evaluating the transferability of their methodologies.

**Table 7:** Appliance level consumption statistics.

Appliance Level Consumption Statistics						
Monitored appliance	A/C	Water Boiler	Dishwasher	Fridge	Washing Machine	Kettle
Total number (#) of monitored appliances	18	12	4	14	13	1
Total Consumption (kWh)	6656	5246	267	4251	1126	69
Total number (#) of appliance activations	2631	5042	346	continuous	1476	73

The statistics include the number of monitored appliances, total consumption, and activations per appliance.

In order to calculate the number of activations of each appliance, we utilized the appliances_metadata.csv, which contains appliance-specific parameters regarding their (1)-wattage, (2)-the wattage threshold for being considered “on”, (3) the minimum and (4) the maximum operational duration. This information was determined through a combination of specifications provided by the manufacturers of the appliances, data analysis, and insights drawn from similar datasets and research studies^27,62,63^. Based on these data, we were able to precisely determine their “on-off” status. The code for the status calculation can be found on the project’s GitHub page.

The environmental dataset includes measurements of indoor and outdoor temperature, as well as humidity levels. The environmental sensors were specifically placed in the living room. The decision to place environmental sensors in the living room was a strategic compromise aimed at capturing a general trend of environmental conditions within the house rather than achieving room-level precision. This approach was chosen under the assumption that the living room’s conditions would broadly reflect the overall environmental changes occurring within the home due to heating or cooling activities. Additionally, upon examining the respective data patterns, it becomes evident that they align with anticipated trends, effectively capturing both seasonal fluctuations as well as the heating and cooling behaviors of the residents.

**Fig. 9:**
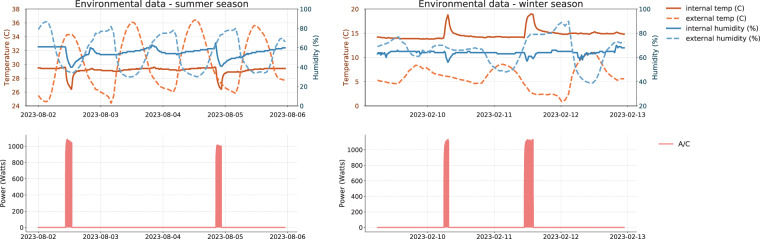
This visualization showcases environmental data collected from House 3 across different seasons, highlighting the heating and cooling practices employed by the residents. The left graph provides insights into environmental conditions in August 2023 and their variations as a result of air conditioning usage for cooling. On the right, we observe environmental data from February, revealing fluctuations driven by the use of air conditioning, which, in this instance, is employed for heating rather than cooling the house.

An illustrative example is depicted in Fig. 9. In this example, the environmental data from House 3 is presented for different time periods, showcasing how these metrics vary with each season (summer and winter) and how heating and cooling practices influence them. On the left side of the chart, which refers to the summer season, we observe that when the A/C is activated for cooling, it leads to a reduction in internal temperature and humidity, as expected. Similarly, in the winter season chart, we observe an increase in internal temperature and a decrease in internal humidity during A/C activation since the A/C was used for heating purposes. This diagram not only validates the environmental data but also highlights their correlation with A/C usage, suggesting potential applications in various contexts. Despite the different granularities—electrical data at 10-second intervals and environmental data every 15 minutes—we observed no significant lags. Temperature and humidity changes occur gradually, enabling effective correlation between the two datasets. Figure 9 supports this, showing a clear match between air conditioning activations and environmental shifts, validating our approach.

### Sociodemographic data & building characteristics

**Fig. 10:**
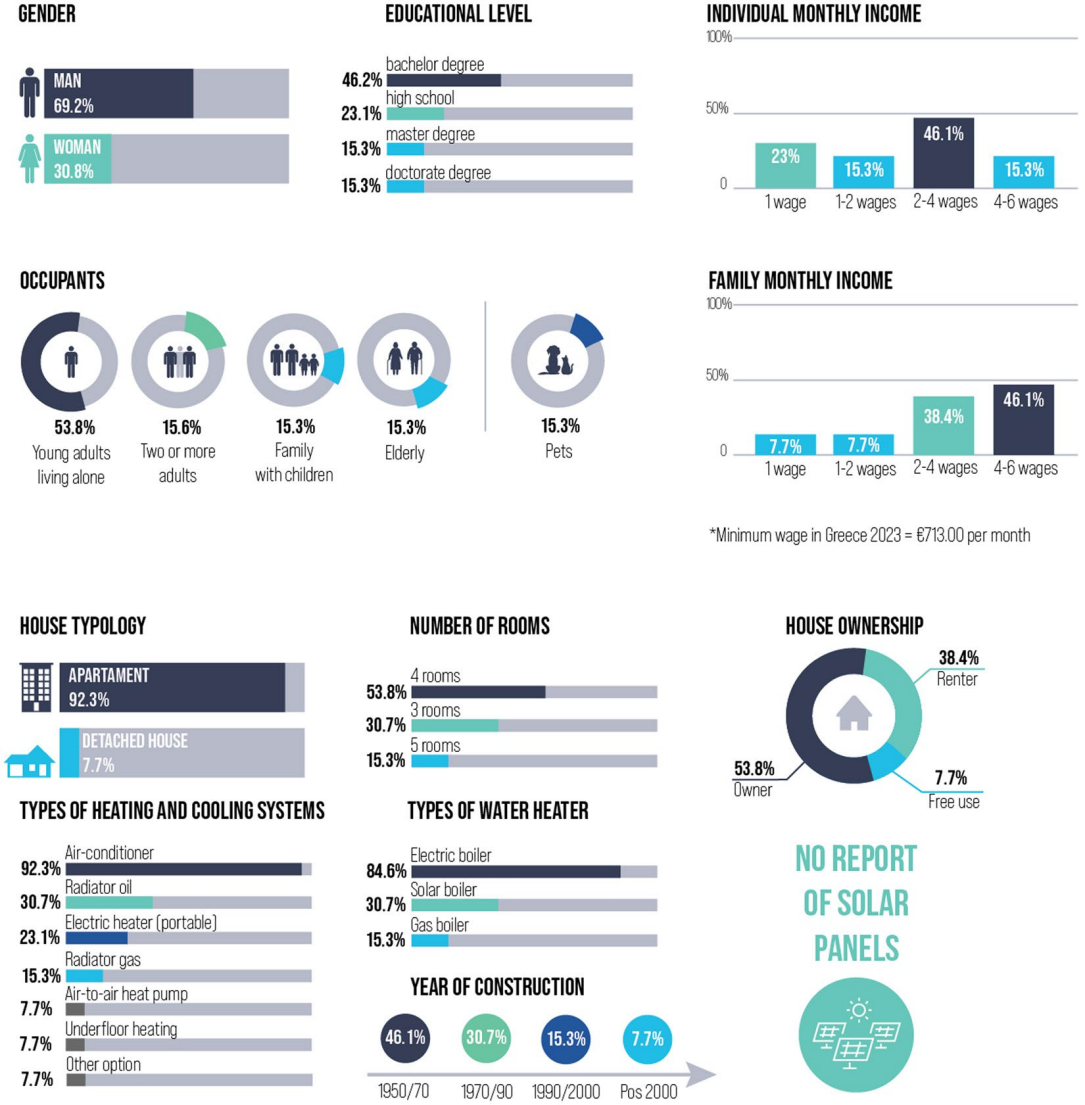
Overview of the sociodemographic and building characteristic data.

At the individual level, 13 respondents answered questions about their gender, educational level and individual monthly income. 69.2% were men in contrast with 30.8% of women. 46.2% has bachelor’s degree, 23.1% high school, 15.3% master’s degree and 15.3% doctorate’s degree. 23% of families earn up to C713.00 per month (up to one minimum wage), 46.1% of recruited households have a monthly income between C1,426.00 and C2,852.00 (equivalent to 2 to 4 minimum wages), and 15.3% of households earn between C2,852.00 and C4,278.00 per month (4 to 6 minimum wages)(see Fig. 10). At the household level, 53.8% are young adults living alone, 15.6% are adults living with two more individuals, 15.3% are families with children, and 15.3% are elderly. 15.3% of households have a pet. The family’s monthly income varies in accordance with the number of occupants in the same house; 46.1% earn between 4 and 6 wages, 38.4% between 2 and 4 wages, 7.7% earn up to 2 wages, and 7.7% earn up to 1 wage.

The predominant house typology is apartments (92.3%) in contrast with only 7.7% detached houses. The number of rooms gives general information about the house size; 53.8% of households have 4 rooms, 30.7% 3 rooms, and 15.3% 5 rooms. In terms of house ownership, 53.8% are owners, 38.4% are renters, and 7.7% have ‘free use’ also described as complimentary occupancy or rent-free living. Regarding the types of heating and cooling systems, most of the households have a combination of two different types. 92.3% have air-conditioner, 30.7% radiator oil, 23.1% electric heater (portable), 15.3% radiator gas, 7.7% air-to-air heat pump, 7.7% underfloor heating and 7.7% declare other options. The predominant water heater is electric boiler (84.6%), following by 30.7% of solar boilers, and 15.3% of gas boilers. No one has declared the ownership of solar panels. Finally, when it comes to year of housing construction, 46.1% were built between 1950 and 1970, 30.7% between 1970 and 1990, 15.3% between 1990 and 2000, and 7.7% post 2000.

Although our dataset may feature participants with educational attainment and monthly incomes exceeding the national median in specific instances, the housing characteristics provided do represent typical living conditions in Attica, where 35% of the Greek population resides^64^. According to Hellenic Statistical Authority (ELSTAT)^64^ 78% of Attica’s housing stock is composed of apartments, with the rest being detached or semi-detached homes. Additionally, the construction years of buildings in Athens predominantly fall between 1950 and 1990, accounting for 63%, while those constructed post-1990 represent 25%, a distribution mirrored in our dataset’s selected households.

## Usage Notes

The data is provided in CSV format and therefore, is usable in most popular software packages, such as MS Excel, Matlab & SPSS, or any other programming language. The Plegma_README file is a valuable resource that details the organization of the dataset, explaining the structure, naming convention, and specific contents of each file, which allows users to locate and utilize the data they need efficiently.

## Supplementary information

Consent Form

Ethical Approval GECKO

Ethical Approval Plegma

Ethical Approval from the ethical committee of NTUA

## Data Availability

The dataset can be efficiently managed, visualized and preprocessed using four Jupyter notebooks. These notebooks are accessible for download at https://github.com/sathanasoulias/Plegma-Dataset To ensure the proper functioning of these notebooks, it is necessary to have Python version 3 along with the Pandas, Plotly and Numpy libraries installed. Moreover, the primary Javascript functions used in the data collection process (Z-wave service and DataBroker service) are located in the data_collection folder giving more details about the implementation of such a system.
