# Author Correction: The Plegma dataset: Domestic appliance-level and aggregate electricity demand with metadata from Greece

**DOI:** 10.1038/s41597-024-03790-3

**Published:** 2024-09-13

**Authors:** Sotirios Athanasoulias, Fernanda Guasselli, Nikolaos Doulamis, Anastasios Doulamis, Nikolaos Ipiotis, Athina Katsari, Lina Stankovic, Vladimir Stankovic

**Affiliations:** 1 National Technical University of Athens, School of Rural, Surveying and Geoinformatics Engineering, Athens, 157 80 Greece; 2 Plegma Labs, Marousi, 151 24 Greece; 3 Aalborg University, Department of the Built Environment, Copenhagen, 2450 Denmark; 4 University of Strathclyde, Department of Electronic and Electrical Engineering, Glasgow, G1 1XQ UK

**Keywords:** Energy efficiency, Energy and behaviour, Technology, Energy management, Energy efficiency, Energy and behaviour, Technology, Energy management

Correction to: *Scientific Data* 10.1038/s41597-024-03208-0, published online 12 April 2024

Figure 10 contained a small inconsistency where the visual representation of percentages did not accurately reflect the numerical data in some cases. This has been updated in the published version with a corrected figure.

