# Supplementary material for: The Plegma dataset: Domestic appliance-level and aggregate electricity demand with metadata from Greece
Source: Sci Data. 2024 Apr 12;11:376. doi: 10.1038/s41597-024-03208-0 (PMC11014970; doi:10.1038/s41597-024-03208-0)
Supplement: Supplementary file 1 — Consent Form [file 41597_2024_3208_MOESM1_ESM.pdf]

Pleg.ma Labs

Participant Information Sheet for Living Labs Data Collection

Introduction
The purpose of collecting data in the Living Lab is to gather electric, demographic building, and environmental data for the purposes of research and innovation. The Marie Skłodowska-Curie GECKO No 955422 project funded the deployment of this project, which covered the costs of equipment installation. Participants did not incur any additional expenses.

What is the purpose of this investigation?
The future of smart buildings involves leveraging sensor data to enhance both the building and occupants' intelligence, leading to actionable outcomes. This entails continuous monitoring of the environment, equipment, systems, processes, and occupants' routines, all aimed at improving living conditions. The data collected in this study will encompass occupant information, aggregate and appliance-level electric consumption data, environmental data, and building-related information. These data will be utilized to develop reliable and effective Connected Home Technology (CHT) applications, including Non-Intrusive Load Monitoring, Demand Response, and Demand Forecasting. Special equipment will be installed in each house to gather this data, which will then be collected and stored in Plegma Labs' central servers. These data play a vital role in advancing CHT applications and driving towards a more energy-efficient society.

Do you have to take part?
Note that your participation is voluntary. Refusing to participate or withdraw participation will not affect any other You can withdraw at any time, for any reason and you do not have to explain your reasons for withdrawing.

What will you do in the project?
If you decide to participate, we will install specialized equipment in your house at no cost. This equipment will collect data on your aggregate and appliance-level electric consumption, as well as environmental data. Additionally, we may request information regarding your demographics, building-related details, and your routine, including domestic appliance usage.

What are the potential risks to you in taking part?
There are no risks in taking part in this study.

What happens to the information in the project?
All data will be de-identified and kept within the Plegma Labs databases. Each participant will be given a unique number, to which data will be referred. The mapping between your unique number and the aforementioned measurements will only be known to Plegma Labs team and will not be disclosed verbally or in writing.

What happens next?
After you have read this information and asked any questions you may have, we will ask you to complete an Informed Consent Form.

The processed results as well as the raw de – identified data may be published in open access journals in the field signal processing and machine learning and will also be used for research and innovation purposes. The identities of participants or exact location of the study will not be disclosed.

Plegma team contact details:
Nikolaos Ipiotis, ni@pleg.ma
Athina Katsari, ak@pleg.ma
Sotirios Athanasoulias, sa@pleg.ma
Michalis Savvakis, ms@pleg.ma
Spiros Chadoulos, sc@pleg.ma
Stelios Kalogridis, stelios@pleg.ma

If you have any questions/concerns, during or after the investigation, or wish to contact an independent person to whom any questions may be directed or further information may be sought from, please contact:

Plegma Labs
Neratziotissis 115 Marousi
Athens
GR-15124

Telephone: +30 210 6109730
Email: info@pleg.ma

Consent Form for Participants

Title of the study: Living Labs Data Collection

- I confirm that I have read and understood the information sheet for the above project and the Plegma team has answered any queries to my satisfaction.
- I understand that my participation is voluntary and that I am free to withdraw from the project at any time, up to the point of completion, without having to give a reason and without any consequences. If I exercise my right to withdraw and I don't want my data to be used, any data which have been collected from me will be destroyed.
- I understand that I can withdraw from the study any personal data (i.e. data which identify me personally) at any time.
- I understand that de-identified data (i.e. data which do not identify me personally) cannot be withdrawn once they have been included in the study.
- I understand that any personal information recorded in the investigation will remain confidential and no information that identifies me will be made publicly available.
- I consent to being a participant in the project.

(PRINT NAME)	
Signature of Participant:	Date:
