# Supplementary material for: The Plegma dataset: Domestic appliance-level and aggregate electricity demand with metadata from Greece
Source: Sci Data. 2024 Apr 12;11:376. doi: 10.1038/s41597-024-03208-0 (PMC11014970; doi:10.1038/s41597-024-03208-0)
Supplement: Supplementary file 2 — Ethical Approval GECKO [file 41597_2024_3208_MOESM2_ESM.pdf]

Universität Siegen
Wirtschaftswissenschaften
Wirtschaftsinformatik
Wirtschaftsrecht

Universität Siegen / Lehrstuhl Wirtschaftsinformatik / 57068 Siegen

Fakultät III /
Lehrstuhl Wirtschaftsinformatik inbs. Datenschutz und IT Sicherheit

US-F 111 (Ludwig-Wittgenstein-Haus)
Kohlbettstr. 15
57068 Siegen

Mail: gunnar.stevens@uni-siegen.de
Telefon: +49 (0) 271 / 740 – 3993
Sekretariat: +49 (0) 271 / 740 – 4036

Ethical Approval for Plegma Labs Data Collection in Alignment with GECKO

This document grants ethical approval for the data collection activities undertaken by Plegma Labs, ensuring full alignment with the ethical standards and regulations set forth by the European Union and the GECKO project, an EU-funded initiative.

Compliance with EU and GECKO Ethics: Plegma Labs commits to adhering to the ethical framework and guidelines established by the European Union and the specific ethical considerations of the GECKO project. This includes, but is not limited to, ensuring participant privacy, data protection, and the ethical use of technology.

Purpose of Data Collection: The data collection efforts by Plegma Labs aim to advance IoT technologies within the scope of the GECKO project's objectives, focusing on innovation, sustainability, and societal benefit.

Voluntary Participation and Informed Consent: Participation in Plegma Labs' data collection is strictly voluntary, with all participants provided with comprehensive informed consent forms. These documents clearly outline the purpose of the data collection, the nature of the data to be collected, and the participants' rights, including withdrawal from the project at any time.

Data Protection and Privacy: In line with GDPR regulations and the ethical guidelines of the GECKO project, Plegma Labs will implement stringent measures to ensure the privacy and protection of all participant data. This includes data anonymization, secure data storage, and limited access to data.

Ethical Oversight: The ethical approval and oversight of Plegma Labs' data collection activities will be conducted in cooperation with the GECKO project's ethics committee, ensuring ongoing compliance with ethical standards.

By this document, the GECKO coordinator hereby approves Plegma Labs' data collection activities, recognizing their alignment with the ethical standards of EU-funded projects and the specific ethics of the GECKO initiative.

Prof. Dr. Gunnar Stevens
(Project coordinator of GECKO)
