# Supplementary material for: The Plegma dataset: Domestic appliance-level and aggregate electricity demand with metadata from Greece
Source: Sci Data. 2024 Apr 12;11:376. doi: 10.1038/s41597-024-03208-0 (PMC11014970; doi:10.1038/s41597-024-03208-0)
Supplement: Supplementary file 3 — Ethical Approval Plegma [file 41597_2024_3208_MOESM3_ESM.pdf]

Pleg.ma
Labs

Industrial Ethical Approval for Data Collection

This document outlines the ethical approval for the data collection project conducted by Plegma Labs S.A., ensuring compliance with the General Data Protection Regulation (GDPR) and ethical standards for research involving human participants.

Purpose of Data Collection: The project aims to enhance Connected Home Technologies for promoting energy efficiency sustainability, and user engagement.

Compliance with GDPR: All data collection processes adhere to GDPR principles, ensuring data protection, privacy, and security. Participants are informed about the data processing activities, data retention periods, and their rights under GDPR.

Voluntary Participation: Participation in this project is entirely voluntary. Participants will be members of Athenian Energy Community and have expressed their interest in contributing to the creation process of Plegma Labs' dataset.

Informed Consent: Participants are provided with a detailed consent form, which includes information about the purpose of the data collection, the types of data collected, how the data will be used, and their rights as participants. The consent form is designed to be clear and understandable, ensuring participants are fully informed before giving their consent.

Access to Data and Platform: Participants are granted real-time access to their collected data through the Plegma Labs platform. Additionally, they will have the opportunity to use and test the Alpha and Beta versions of the platform, providing valuable feedback for its development.

Data Protection and Privacy: Measures are in place to protect the privacy and security of participants' data. This includes anonymizing data where possible, secure data storage, and restricted access to data.

Plegma Labs S.A. is committed to conducting its data collection project with the utmost respect for ethical principles, data protection laws, and the welfare of participants. We believe that this project will contribute significantly to the advancement of IoT technologies while ensuring the privacy and rights of our participants are protected.

Nikos Ipiotis

CEO of Plegma Labs

PLEGMA LABS A.E.
ΤΕΧΝΟΛΟΓΙΚΕΣ ΛΥΣΕΙΣ
ΛΕΩΦΟΡΟΣ ΚΗΦΙΣΙΑΣ 227, ΚΗΦΙΣΙΑ 145 61
ΑΦΜ: 800512150 - ΔΟΥ: ΦΑΕ ΑΘΗΝΩΝ
Αρ. ΓΕΜΗ: 126533701000

Athina Katsari

Data Protection Officer
