# Supplementary material for: The Plegma dataset: Domestic appliance-level and aggregate electricity demand with metadata from Greece
Source: Sci Data. 2024 Apr 12;11:376. doi: 10.1038/s41597-024-03208-0 (PMC11014970; doi:10.1038/s41597-024-03208-0)
Supplement: Supplementary file 4 — Ethical Approval from the ethical committee of NTUA [file 41597_2024_3208_MOESM4_ESM.pdf]

ΑΠΟΦΑΣΗ

ΕΠΙΤΡΟΠΗΣ ΗΘΙΚΗΣ ΚΑΙ ΔΕΟΝΤΟΛΟΓΙΑΣ ΤΗΣ ΕΡΕΥΝΑΣ (Ε.Η.Δ.Ε.) ΤΟΥ ΕΘΝΙΚΟΥ ΜΕΤΣΟΒΙΟΥ ΠΟΛΥΤΕΧΝΕΙΟΥ

ΓΙΑ

ΕΓΚΡΙΣΗ ΕΡΕΥΝΗΤΙΚΟΥ ΠΡΩΤΟΚΟΛΛΟΥ

ΕΜΠΙΣΤΕΥΤΙΚΟ ΕΓΓΡΑΦΟ

Τίτλος μελέτης για την οποία ζητήθηκε έγκριση

«Plegma dataset» στο πλαίσιο του έργου GECKO με ΚΑ 63/2350

Επιστημονικός Υπεύθυνος της μελέτης

Δουλάμης Νικόλαος (Καθηγητής ΕΜΠ)

Είδος προτεινομένης μελέτης

Το σύνολο δεδομένων Plegma παρέχει μετρήσεις συνολικής αλλά και κατανάλωσης σε επίπεδο συσκευής σε διαστήματα 10 δευτερολέπτων από 13 διαφορετικά νοικοκυριά κατά τη διάρκεια ενός έτους, ξεκινώντας τον Ιούλιο του 2022. Το σύνολο δεδομένων Plegma παρέχει συνολικά φορτία ολόκληρου του σπιτιού και μετρήσεις κατανάλωσης σε επίπεδο συσκευής σε διαστήματα 10 δευτερολέπτων από 13 διαφορετικά νοικοκυριά κατά τη διάρκεια ενός έτους, ξεκινώντας τον Ιούλιο του 2022. Περιλαμβάνει επίσης περιβαλλοντικά δεδομένα όπως η υγρασία και η θερμοκρασία, χαρακτηριστικά των κτιρίων, δημογραφικές πληροφορίες και συνήθειες των χρηστών για να επιτρέψουν τόσο ποσοτικές όσο και ποιοτικές αναλύσεις. Το σύνολο δεδομένων περιλαμβάνει 218 εκατομμύρια αναγνώσεις από 88 εγκατεστημένους μετρητές και αισθητήρες. Τα συλλεγόμενα δεδομένα έχουν καθαριστεί για να χειριστούν τα απουσιάζοντα δεδομένα και τις λανθασμένες μετρήσεις, και είναι διαθέσιμα σε μορφή αρχείων CSV.

Αριθμός Πρωτοκόλλου Ε.Η.Δ.Ε./ Αριθμός Πρωτοκόλλου Ε.Λ.Κ.Ε.

15623/20.03.2024

Αριθμός & Ημερομηνία Απόφασης Επιτροπής Ηθικής και Δεοντολογίας της Έρευνας (Ε.Η.Δ.Ε.)

Συνεδρίαση 29.03.2024, Θέμα 1.1

Απόφαση Επιτροπής Ηθικής και Δεοντολογίας της Έρευνας (Ε.Η.Δ.Ε.)

Εγκρίνεται

Μέλη της Επιτροπής

Α. Ανδρεόπουλος (Πρόεδρος), Δ. Σούντρης, Ε. Παυλάτου, Π. Στεφανέας, Δ. Μαμάης, Λ. Αλεξόπουλος

Σχόλια από την Επιτροπή Ηθικής και Δεοντολογίας της Έρευνας (Ε.Η.Δ.Ε.) με βάση τα οποία λήφθηκε η απόφαση για την αίτηση που υποβλήθηκε

Μελετώντας το ερευνητικό πρωτόκολλο και όλα τα σχετικά δικαιολογητικά/πρόσθετες εγκρίσεις, όπως κατατέθηκαν στην Επιτροπή Ηθικής και Δεοντολογίας της Έρευνας (Ε.Η.Δ.Ε.),

και

λαμβάνοντας υπόψη τους σκοπούς και τα αναμενόμενα οφέλη, τη μεθοδολογία της έρευνας, την έλλειψη σύγκρουσης συμφερόντων από τους ερευνητές και την έλλειψη

πιθανών κινδύνων για τα υποκείμενα της έρευνας, σύμφωνα με τα διαλαμβανόμενα στη σχετική εισηγητική έκθεση,

η ΕΗΔΕ

διαπιστώνει και ομόφωνα εγκρίνει την υποβληθείσα αίτηση (άρθρο 279 παρ. 1 ν. 4957)

Η παρούσα απόφαση της ΕΗΔΕ σε καμία περίπτωση ΔΕΝ υποκαθιστά την απαιτούμενη από άλλη αρμόδια δημόσια υπηρεσία, διοικητικό όργανο ή ανεξάρτητη διοικητική Αρχή, έγκριση ή αδειοδότηση του παρόντος ερευνητικού έργου/ μελέτης που δύναται επιπλέον να απαιτείται εκ του νόμου.

Ημερομηνία έκδοσης απόφασης		
Έτος: **2024**	Μήνας: **Μάρτιος**	Ημέρα: **29, Παρασκευή**

Υπογράφει ο Πρόεδρος της Επιτροπής			
Θέση	Όνομα	Επώνυμο	Υπογραφή
**Πρόεδρος**	Ανδρέας	Ανδρεόπουλος	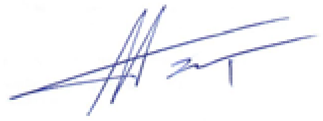
